# Cerebral Radiation-Induced Vasculopathy Mimicking Primary Central Nervous System Vasculitis

**DOI:** 10.7759/cureus.43659

**Published:** 2023-08-17

**Authors:** Haifa K Abdulghaffar, Abdulaziz A Alqarni, Samaher Altwirgi, Bader Shirah, Manal Badawi, Ahmed Hassan

**Affiliations:** 1 College of Medicine, King Saud bin Abdulaziz University for Health Sciences, Jeddah, SAU; 2 College of Medicine, Royal College of Surgeons in Ireland, Dublin, IRL; 3 Department of Neuroscience, King Faisal Specialist Hospital and Research Centre, Jeddah, SAU

**Keywords:** seizures, stroke, primary central nervous system vasculitis, cranial irradiation, radiation-induced vasculopathy

## Abstract

Cranial irradiation is one of the main treatment modalities for tumors of the CNS. However, it can lead to significant damage to the treated region. Among the late complications of radiation therapy to the brain is vasculopathy of the small and/or large arteries. In this article, we report a case of CNS radiation-induced vasculopathy presenting 30 years after cranial irradiation and mimicking primary CNS vasculitis. The present case illustrates the importance of monitoring and carefully evaluating delayed side effects of radiotherapy as well as emphasizes the importance of obtaining a detailed history of any patient presenting with sudden unexplained symptoms. If a complete proper history of the patient’s past medical diagnoses and procedures was taken, medical professionals would not have needed to conduct extensive investigations and implement treatment plans for a less likely diagnosis, in this case, aggressive treatment of a possible primary CNS vasculitis with high-dose steroids. Therefore, it is imperative to raise the possibility of radiation-induced vasculopathy after excluding all possible causes of deterioration in patients with a history of cranial radiation therapy.

## Introduction

Cranial irradiation is one of the main treatment modalities for tumors of the CNS. However, it can lead to significant damage to the treated region. Among the late complications of radiation therapy to the brain is vasculopathy of the small and/or large arteries. The period between exposure to radiation and the development of vasculopathy typically ranges from 2 to 25 years [1]. Neuroradiological studies using CT and MRI do not accurately predict the occurrence of brain damage caused by radiation exposure [2]. The majority of patients who undergo radiotherapy for brain tumors present with focal ischemic episodes, while other symptoms, such as headaches, impaired consciousness, vertigo, and seizures, are less common [3]. In this article, we report a case of CNS radiation-induced vasculopathy presenting 30 years after cranial irradiation and mimicking primary CNS vasculitis.

## Case presentation

**Figure 1:**
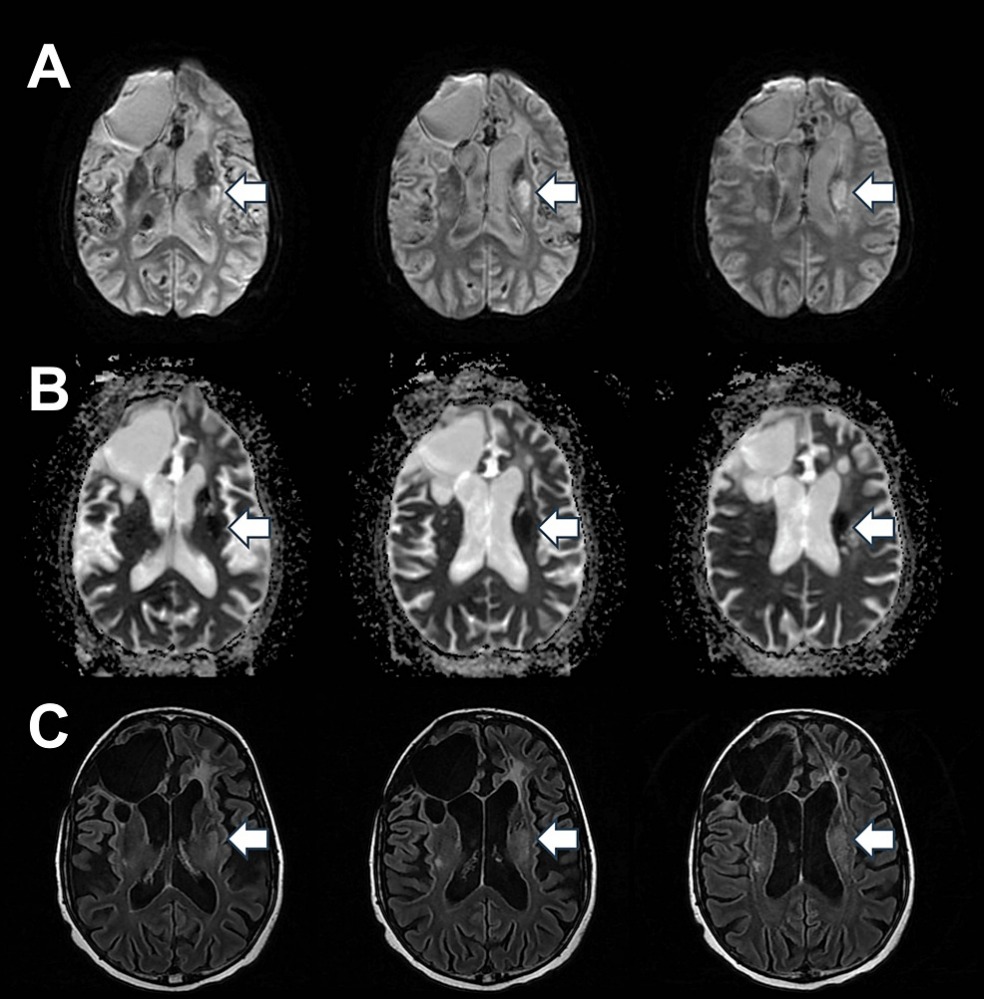
Brain MRI with (A) diffusion-weighted imaging, (B) apparent diffusion coefficient, and (C) fluid-attenuated inversion recovery sequences showing acute left corona radiata/caudate body stroke extending to the external capsule (arrows)

A 41-year-old male, with a known case of hypertension, obesity, and recurrent seizures that started following a brain tumor resection and extensive brain radiotherapy at the age of 12 years old, presented to the emergency room complaining of right-sided weakness involving the face, arm, and leg associated with slurred speech. Physical examination revealed expressive aphasia with non-fluent speech and paraphasic error, dysarthria, right-sided facial palsy, and weakness in the right arm and leg (no effort against gravity). His NIH stroke scale score was 12. Brain MRI revealed acute left corona radiata/caudate body ischemic stroke extending to the external capsule (Figure 1).

The patient was admitted for a “stroke-in-young” workup and was started on aspirin 81 mg. Brain MR angiography showed atherosclerotic changes in the cavernous segments of the internal carotid arteries causing a mild degree of stenosis as well as focal areas of stenosis in the P1 segment of the left posterior cerebral artery and P2 segment of the right posterior cerebral artery. Echocardiogram, 24 h Holter monitoring, and hypercoagulable panel were unremarkable. Hence, the patient was then discharged on aspirin 81 mg. At that time, he was on three anti-seizure medications (lacosamide, lamotrigine, and valproic acid).

**Figure 2:**
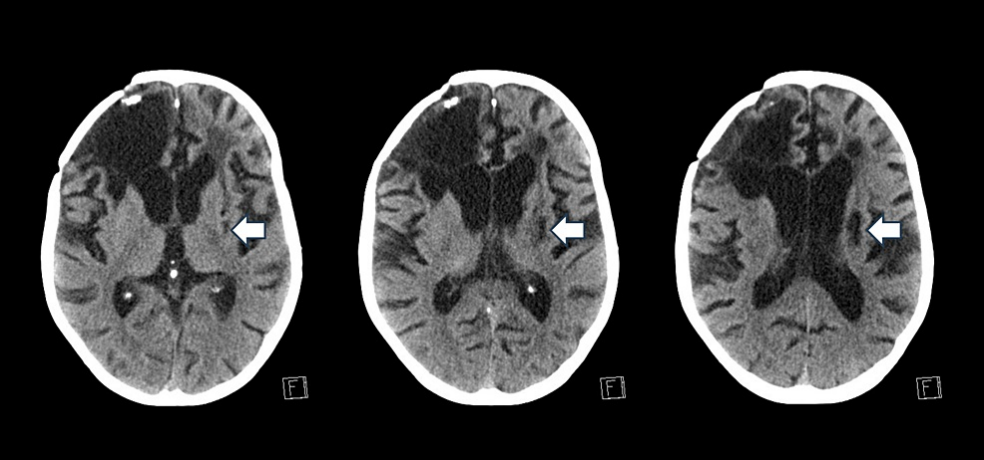
CT brain showing old infarction in the left corona radiata and basal ganglia hypodensities extending to the left external capsule with associated ipsilateral Wallerian degeneration (arrows)

One month later, the patient presented to the emergency room with impaired awareness, uprolling of the eyes, head deviation to the right, and jerky movements of the limbs that lasted for 15 minutes that were aborted after lorazepam 2 mg and a loading dose of levetiracetam 1500 mg intravenously, and then the patient returned to baseline. He was admitted as a case of status epilepticus for further investigation, as the family also reported increased seizure frequency and duration, and choking attacks. Brain CT done after the resolution of the seizures showed no acute brain insult. EEG showed diffuse theta slowing with further sharp slowing of moderate voltage in the right hemisphere. During admission, the patient had multiple breakthrough seizures which were aborted by lorazepam 2 mg and levetiracetam 1500 mg. He had fluctuations in his level of consciousness for which EEG was repeated and showed evidence of continuous and diffuse disturbances of cerebral activities with diffuse slowing of the background activity in the 5-6 Hz theta range with polymorphic morphology, right temporal and parasagittal slowing, and asymmetry in amplitude (right higher than left) indicative of diffuse cerebral insult as well as further pathology in the right hemisphere, which is epileptogenic in nature. Due to the unexplained nature of his symptoms, extensive investigations were performed, including routine hematologic tests, including complete blood count, coagulation profile, chemistry, hormones, tumor markers, toxicology, urine workup, CSF analysis, microbiology, serology, autoimmune workup, complements, whole exome sequencing, and imaging of the brain as well as CT chest, abdomen, and pelvis with contrast, thyroid ultrasound, and scrotal ultrasound to exclude possible malignancy. The paraneoplastic workup was negative. CT brain showed old infarction in the left corona radiata and basal ganglia hypodensities extending to the left external capsule with associated ipsilateral Wallerian degeneration (Figure 2).

**Figure 3:**
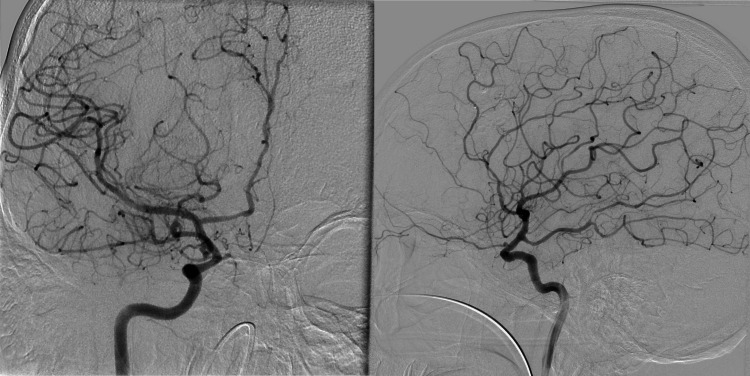
Conventional cerebral angiography showing narrowings affecting the middle cerebral arteries and the anterior cerebral arteries bilaterally

After excluding multiple possible explanations for this clinical presentation, the patient was suspected to have primary CNS vasculitis vs. CNS radiation-induced vasculopathy. He underwent an MRI vessel wall, but it was degraded by motion artifacts and non-contributory. In addition, meningeal biopsy and autoimmune workup yielded negative results. Conventional cerebral angiography showed multifocal narrowings affecting the middle cerebral arteries and the anterior cerebral arteries bilaterally (Figure 3).

CSF analysis showed high protein 522 mg/L, high RBC 24 10^ 6/L, normal WBC, and normal glucose. The patient was then started on a course of methylprednisolone 1000 mg for five days with significant improvement regarding his level of consciousness.

A more detailed history was sought from the family. At the age of 12 years, the patient had a history of headaches for which he sought medical care and was then diagnosed as having a right frontal lobe brain mass. Subsequently, he underwent a brain tumor resection and received 27 sessions of brain radiotherapy. Afterward, the patient started experiencing multiple seizure attacks, in addition to progressively deteriorating cognitive function. During that period of time, the patient was discharged with carbamazepine to control his seizures. He was compliant with the medication and was satisfied with the results until he stopped it at the age of 15 years old, and the seizures resumed. His seizures were focal, non-motor with impaired awareness.

The suspicion of radiation-induced vasculopathy was thoroughly debated, considering the fact that the radiotherapy sessions were given almost 30 years ago and the disadvantages and/or limitations of such treatment modality were unknown at the time. In addition, information regarding the exact diagnosis, type of brain tumor, and type of radiotherapy used 30 years ago was unknown to the family, and no medical reports were available. However, it was concluded that the final diagnosis that may explain this unusual neurological presentation is radiation-induced vasculopathy considering that symptoms started immediately after receiving the 27 sessions of radiotherapy and after excluding all other possible cases of these manifestations.

## Discussion

Radiation-induced vasculopathy was first described in 1967 by Lee and Hodes [4]. It is primarily caused by tissue necrosis and inflammation [5]. Small vessels become necrotic as a result of endothelial cell damage directly caused by radiation cytotoxicity. As a consequence of hypoxia, other vessels over the corresponding areas are damaged. A disruption of the blood-brain barrier may result in diffuse atherosclerosis, hyalinization, and even micro-bleeding months to years following irradiation of the brain [6]. Studies showed that injuries to small vessels appear earlier than injuries to large arteries [7].

After radiation, signs and symptoms may appear four months to 24 years later. Symptoms of this entity are not pathognomonic but appear similar to those seen in cerebral infarction, transient ischemic attack, and moyamoya disease [8]. Numerous studies indicated that primary moyamoya syndrome and progressive cerebral vasculopathy, including radiation-induced vasculopathy, share common pathophysiological mechanisms. Radiologically, radiation-induced vascular changes are not distinguishable from primary moyamoya syndrome [9]. Patients receiving adjunctive chemotherapy, receiving radiotherapy at a young age, receiving a higher radiation dose, or having other vascular risk factors are among the risk factors for developing radiation-induced vasculopathy [10].

Generally, radiation-induced vasculopathy is a diagnosis of exclusion, in a case with a history of radiation therapy to the affected vessels. Patients should be tested for conditions that could predispose them to vasculopathy including vasculitis, collagen vascular disease, atherosclerosis, neurofibromatosis, primary moyamoya syndrome, and other causes of occlusive vasculopathy, such as tumor compression, diabetes, and hypertension [11,12].

There is a lack of details in most reports regarding radiation therapy doses and techniques, resulting in an unclear dose-response relationship and volume effect. Radiation-induced vasculopathy most commonly occurs in patients who have been exposed to radiation in the parasellar region. After irradiation with doses of 10-105 Gy, most patients experience their first symptoms of this cerebrovascular disorder within eight years [8,11].

Our patient developed an ischemic stroke and worsening seizures 30 years after cerebral irradiation. Radiation-induced vasculopathy was diagnosed based on radiological criteria and after ruling out all other possible causes. In view of the fact that all neuroimaging scans did not reveal any evidence of vascular compression caused by tumor recurrence, and no clinical signs of a neurocutaneous syndrome were present, the observed vasculopathy was attributed to long-term complications associated with cranial radiation therapy.

## Conclusions

We described a case of CNS radiation-induced vasculopathy in a young Saudi male who developed a stroke and increased seizure frequency 30 years following 27 cerebral radiation therapy sessions. The present case illustrates the importance of monitoring and carefully evaluating delayed side effects of radiotherapy as well as emphasizes the importance of obtaining a detailed history of any patient presenting with sudden unexplained symptoms. It is imperative to raise the possibility of radiation-induced vasculopathy after excluding all possible causes of deterioration in patients with a history of cranial radiation therapy.
